# Can Algal Derived Bioactive Metabolites Serve as Potential Therapeutics for the Treatment of SARS-CoV-2 Like Viral Infection?

**DOI:** 10.3389/fmicb.2020.596374

**Published:** 2020-11-11

**Authors:** Ankita Bhatt, Pratham Arora, Sanjeev Kumar Prajapati

**Affiliations:** ^1^Environment and Biofuel Research Lab, Department of Hydro and Renewable Energy, Indian Institute of Technology Roorkee, Roorkee, India; ^2^Department of Hydro and Renewable Energy, Indian Institute of Technology Roorkee, Roorkee, India

**Keywords:** microalgae, seaweed, antiviral, COVID-19, sulphated polysaccharides

## Introduction

Microalgae are defined as photosynthetic and unicellular organisms that demonstrate a wide range of adaptability to adverse environmental conditions like temperature extremes, photooxidation, high or low salinity, and osmotic stress (Holzinger and Karsten, 2013; Singh et al., 2019). Alternatively, macroalgae or seaweed includes multicellular, macroscopic, and marine algae belonging mostly to two phyla, namely, Rhodophyta and Phaeophyta (Peng et al., 2015). The micro/macro-algae have recently emerged as a source of various bioactive compounds like phycocyanin, lutein, vitamin E, B12 and K1, polyunsaturated fatty acids, polysaccharides and phenolics (Peng et al., 2015; Costa et al., 2020). These secondary metabolites have been studied for their anti-microbial, anti-inflammatory, immunosuppressive, anti-cancer and other pharmacologically important activities (Sathasivam et al., 2019). Thus, the algal metabolites find wide applicability in a vast array of biotechnological and pharmaceutical fields.

In view of the ongoing COVID-19 pandemic caused by a novel coronavirus, designated as Severe Acute Respiratory Syndrome Coronavirus 2 (SARS-CoV-2), increased efforts are being made for developing efficient treatment options to tackle the disease. The SARS-CoV-2 has been identified as a single-stranded, positive-sense RNA virus belonging to the *Betacoronavirus* family (Yeo et al., 2020). Further, various structural (spike glycoprotein), non-structural (3-chymotrypsin-like protease, helicase, papain-like protease, and RNA-dependent RNA polymerase), and accessory proteins are encoded by SARS-CoV-2 genome (Li and De Clercq, 2020). The spike glycoprotein has been considered to be involved in the interaction between viruses and receptors present on the host cell (Li and De Clercq, 2020). Since this glycoprotein is an essential requirement for the entry of virus in host cells, many recent studies are focused on this structural protein (Zumla et al., 2016). It has been further concluded that the above mentioned five proteins also emerged as attractive targets for antiviral studies against SARS (Severe Acute Respiratory Syndrome) and MERS (Middle East respiratory syndrome) (Zumla et al., 2016).

Considering all the facts related to exploration of algae for bioactive molecules, the present study provides an insight into the utilization of micro/macro-algal metabolites as therapeutic compounds against SARS-CoV-2 and like viruses. The key antiviral metabolites, namely, phycocyanobilins, lectins, and, sulphated polysaccharides have been discussed.

## Algae; A Treasure-trove of Bioactive Metabolites

### Phycocyanobilins; The Antiviral Chromophores

**Figure 1:**
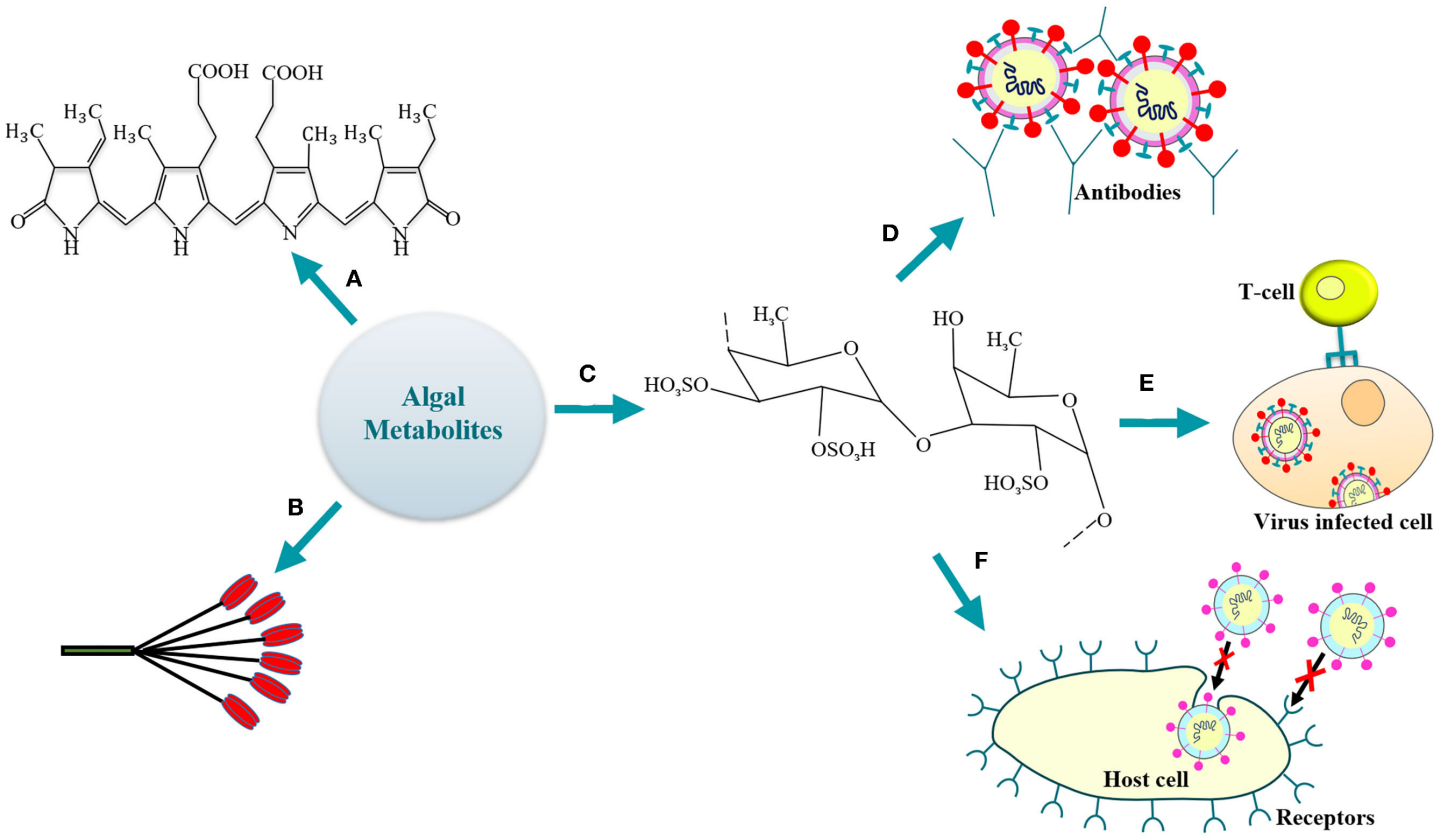
Algal antiviral metabolites (a) phycocyanobilin; (b) lectin; (c) fucoidan (a representative SP); (d) SPs-mediated humoral activation; (e) activation of host cellular immune response by SPs; (f) SPs-driven inhibition of virus entry/attachment to the host cell receptor.

Phycocyanobilins (PCBs) are tetrapyrrole chromophores present in certain cyanobacteria, rhodophytes, and are classified as blue phycobilinis (Figure 1) (Guedes et al., 2019). These light-capturing pigments are now widely studied for their antioxidative, antiviral (Hirata et al., 2000; Ramakrishnan, 2013) and NADPH-oxidase inhibitory activity (McCarty, 2007). Recently, Pendyala and Patras (2020) discussed the possible utilization of PCBs (source—*Spirulina* sp.) as inhibitors for the SARS-CoV-2 infection. The study involved *in-silico* screening (by the COVID-19 Docking Server) of the bioactive compounds for their activity against SARS-CoV-2. It was observed that the phycocyanobilin demonstrates a high binding affinity toward the potential targets, namely, the Main protease (Mpro) and RNA-dependent RNA polymerase (RdRp). The Main protease is involved in the processing of polyproteins (translated from SARS-CoV-2 RNA) while the replication of viral RNA is catalyzed by the polymerase. High binding energy of −8.6 kcal/mol was observed for PCB-Mpro while −9.3 kcal/mol for PCB-RdRp. Noteworthy, the PCB demonstrated a superior binding to target enzymes as compared to antiviral drugs like remdesivir (−8.1 kcal/mol for Mpro, −9.0 kcal/mol for RdRp), lopinavir (−7.9 kcal/mol) and nelfinavir (−7.9 kcal/mol for Mpro, −9.3 kcal/mol for RdRp). Thus, the study highlighted the significant potential of PCB as antiviral. However, as recommended by Pendyala and Patras (2020), further *in-vitro* and/or *in-vivo* studies will be crucially needed to support the obtained docking results and unravel the underlying potential of PCB as therapeutic for COVID-19. Additionally, the purified allophycocyanin obtained from *Spirulina platensis* has been demonstrated to exhibit significant activity against enterovirus 71 (Singh et al., 2020). It was observed that the cytopathic effects of the viral infection were neutralized and the viral RNA synthesis was delayed by the microalgal pigment allophycocyanin. Likewise, results of an *in-silico* study reported that the PCB expressed by *Arthrospira* sp. could serve as a potent antiviral against SARS-CoV-2 (Petit et al., 2020). The study evaluated the interaction between the *Arthrospira* sp. PCB and the receptor binding domain (RBD) of SARS-CoV-2 spike glycoprotein. It was observed that five Van der Waals interactions (involving residues ARG403, TYR453, LEU492, GLN493, and ASN501) contributed to the PCB/Spike RBD complex. The five π-alkyl bonds between the PCB and spike RBD involved the residues TYR449, TYR495, PHE497, and TYR505 with a hydrogen-bond on TYR449. The other residues involving the hydrogen-bond were SER494, GLY496, and GLN498 with the GLY496 linked to PCB by a π-donor hydrogen bond. Finally, a competitive binding energy (−7.2 kcal/mol) demonstrated the possibility to employ PCB as a potential antiviral agent (Petit et al., 2020). A recent study also reported the probability to utilize phycocyanobilin containing cyanobacteria like *Spirulina* sp. to control the RNA virus infections (Nikhra, 2020). A decrease in mortality rate in influenza-infected mice has been observed when administered orally with phycocyanin rich cold-water *Spirulina* sp. extracts in animal experimentation studies. The cold-water extract was well-tolerated even at high concentrations of 3,000 mg/kg/day in animal models for a period of 14 days (Chen et al., 2016). The PCB extracts thus demonstrated a substantial reduction in the survival of zoonotic RNA viruses by enhancing the type 1 interferon response of host immune system (Nikhra, 2020). Hence, it is likely possible that PCB producing microalgae may demonstrate substantial activity against SARS-CoV-2 as well (Cascella et al., 2020; Zhou et al., 2020). Moreover, further research along with *in-vivo* studies is necessary to understand the specific bioactivity of PCBs for the development of therapeutic strategies against human pathogenic viruses, including SARS-CoV-2.

### Lectins; Potential Therapeutic Against SARS-CoV-2

The macroalgae are rich in certain carbohydrate-binding proteins called lectins that demonstrate high specificity for sugar groups of other molecules like the oligosaccharide chains of the viral glycoproteins (Figure 1). Thus, lectins have been widely employed in various pharmacological and medical applications (Breitenbach Barroso Coelho et al., 2018). The mannose-binding lectins (MBL) are the predominant proteins to be studied in the viral infection pathways (Mitchell et al., 2017). The self-assembly of viruses during replication is interrupted by MBLs (Liu et al., 2015); thus, they have also emerged as a potential therapy against Ebola (Michelow et al., 2011).

The red algae-derived lectins were initially brought to the limelight when griffithsin was discovered by Watson and Waaland (1983) from *Griffithsia* sp. Since then, it has been widely studied for various applications (Mori et al., 2004). It has been observed to possess high specificity for mannose residues present on viral glycoproteins. Some studies have demonstrated its antiviral activity against HIV-1 (Lusvarghi et al., 2016), Hepatitis C (Meuleman et al., 2011), and SARS-CoV glycoprotein (Zumla et al., 2016). A recent study analyzed the anti MERS-CoV activity of griffithsin and concluded that the lectin inhibits the entry of the virus while imparting negligible cellular toxicity (Millet et al., 2016). The inhibitory effect of griffithsin at the binding step during virus infection was assayed by time-course experiments. Thus, the study by Millet et al. (2016) demonstrated the griffithsin-mediated inhibition of MERS-CoV infectivity *in*-*vitro*. Additionally, various studies have reported the *in-vivo* antiviral activity of griffithsin against Japanese encephalitis virus (Ishag et al., 2013), herpes simplex virus 2 (Nixon et al., 2013) and human papillomavirus (Levendosky et al., 2015). For instance, the impact of an anti-HIV griffithsin containing microbicide on the rectal microbiome was assessed in the non-human primates (*Rhesus macaques*) (Girard et al., 2018). It was observed that 0.1% of griffithsin gel did not negatively impact the rectal mucosal proteome or microbiome. Further, O'Keefe et al. (2010) reported a 100% survival of model mice infected with a high dose of SARS-CoV upon providing a griffithsin dose of 10 mg/kg(b.w.)/day. Based on griffithsin activity against SARS-CoV, it may be investigated as a therapeutic option for SARS-CoV-2.

Likewise, a novel D-mannose-binding lectin was identified from the red macroalgae *Grateloupia chianggi* and designated as GCL (*Grateloupia chianggi* lectin) (Hwang et al., 2020). The study focussed on GCL purification, its molecular and functional characterization, and subsequent analysis of its antiviral activity against influenza virus, herpes simplex virus and HIV. A quantity of 1–20 nM GCL was required for effective inhibition of HSV. Thus, it may be concluded that GCL also holds the potential to be utilized in virology and biomedical research. It is significant to note here that the SARS-CoV-2 is similar to the influenza virus as both are characterized as enveloped RNA viruses (Noda, 2012; Yeo et al., 2020). Based on the activity of GCL against the influenza virus, its activity may be explored against SARS-CoV-2 as well.

### Sulphated Polysaccharides; Favorable Antiviral Candidates

Various researchers have demonstrated the beneficial effects of algal sulphated polysaccharides (SPs) under defined *in-vitro* and/or *in-vivo* conditions. Both the cellular and/or the humoral response of the immune system can be activated by these compounds (de Paniagua-Michel et al., 2014) (Figure 1).

A recent study emphasized on the purification and structural characterization of two fucoidans from the brown macroalgae *Sargassum henslowianum* (Sun et al., 2020). These fucoidans designated as SHAP-1 and SHAP-2 were studied for their activity against two strains of herpes simplex virus, i.e., HSV-1 and HSV-2. It was observed that both compounds possessed significant anti-HSV activity with the IC_50_ value estimated to be 0.89 and 0.82 μg/mL for SHAP-1 and SHAP-2, respectively, against HSV-1 strain. Surprisingly, the IC_50_ values for both polysaccharides against HSV-2 were very low, i.e., 0.48 μg/mL. Also, time-of-addition experiments revealed that more efficient anti-HSV activities were obtained when fucoidans were added during the infection stage, thereby signifying their role at the early stages of viral infection. The adsorption and penetration assays further demonstrated that the fucoidans were involved in interruption of HSV adsorption to the host cell. Hence, it may be concluded that fucoidans could serve as promising candidates for inhibition of HSV-2 viruses and may be successfully utilized for various clinical applications. Similarly, a sulphated polysaccharide was isolated from the green macroalgae *Monostroma nitidum* (Wang et al., 2020). The compound isolated from *M. nitidum* was identified as a water-soluble sulphated glucuronorhamnan and thus designated as MWS. Various cytotoxicity and antiviral assays were performed to estimate the activity of MWS against EV71, a strain of human pathogenic enterovirus. It was observed that MWS was not toxic to the used cell lines and demonstrated a broad-spectrum of antiviral activity, especially against EV71 under defined *in-vitro* conditions. Further, it was concluded that MWS inhibits the EV71 infection by either targeting the host signaling pathway (down-regulation of host phosphoinositide 3-kinase/protein kinase B signaling pathway) in EV71 early life cycle and/or interrupting adsorption of virus to the host cell. The former mechanism has been concerned with the suppression of viral infection. The study also involved animal experiments, and a significant reduction in the viral titers was observed upon intramuscular administration of MWS in EV71 infected mice (Wang et al., 2020). Additionally, the SPs obtained from macroalgae *Cladosiphon okamuranus* and *Ulva clathrata* were also observed to demonstrate significant antiviral activity against the Newcastle disease virus under defined *in-vitro* conditions (Aguilar-Briseño et al., 2015). Another study elaborated the antiviral activity of SPs obtained from *Ulva pertusa, Grateloupia filicina*, and *Sargassum qingdaoense* against the avian influenza virus under *in-vitro* and *in-vivo* conditions (Song et al., 2016).

A recent review highlighted the possibility of utilizing the SPs obtained from *Porphyridium* sp. (red microalga) as a potential therapeutic to combat COVID-19 disease (Gaikwad et al., 2020). Based on the antiviral activity of *Porphyridium* polysaccharides against a wide range of viruses including HSV (Huheihel et al., 2002), varicella zoster virus (Raposo et al., 2013), hepatitis B virus, vaccinia virus (Radonić et al., 2010) and retroviruses (Xiao and Zheng, 2016), this microalga has been considered to hold immense potential for the development of an antiviral pharmaceutical composition against SARS-CoV-2 as well (Gaikwad et al., 2020). Also, the effective inhibition (*in-vitro*) of SARS-CoV-2 by SPs (fucoidans) obtained from macroalgae *Saccharina japonica* was reported by Kwon et al. (2020). The fucoidans labeled as RPI-27 and RPI-28 demonstrated significant activity against SARS-CoV-2 with RPI-27 being more potent than the antiviral drug remdesivir. These highly branched fucoidans were observed to interfere with the binding of viral S protein to the heparan sulfate co-receptor of the host cells, thereby, inhibiting the viral infection. Thus, the study suggested the possibility of utilizing fucoidans alone or in combination with other antivirals as a promising therapeutic strategy against SARS-CoV-2 infection (Kwon et al., 2020). These studies indicate the potential therapeutic role of algal sulphated polysaccharides.

## Discussion and Conclusion

There has been a substantial increase in evidence that reveals the antiviral activity of various microalgal and macroalgal metabolites like lectins, sulphated polysaccharides, and phycocyanobilins. Recent studies have reported that these compounds demonstrate substantial activity against a wide array of DNA and RNA viruses, including the influenza virus known to be associated with respiratory illnesses. As discussed, the bioactive molecules could serve as a novel therapeutic option to tackle SARS-CoV-2 and alike viruses. Considering the dire need for the development of therapeutics against SARS-CoV-2, there is a necessity to screen through the myriad of algae-derived potential antivirals which demands further evaluation and research.

## Author Contributions

AB: conceptualization, data curation, visualization, and writing - original draft. PA: validation, writing - review & editing. SP: conceptualization, writing - review & editing, and supervision. All authors contributed to the article and approved the submitted version.

## Conflict of Interest

The authors declare that the research was conducted in the absence of any commercial or financial relationships that could be construed as a potential conflict of interest.
